# Community sport organizations, regional governance, and sport policy implementation in Ontario, Canada

**DOI:** 10.3389/fspor.2025.1549966

**Published:** 2025-06-10

**Authors:** Kyle A. Rich, Dante Losardo

**Affiliations:** Faculty of Applied Health Sciences, Brock University, St Catharines, ON, Canada

**Keywords:** sport policy, policy implementation, community embeddedness, regional governance, sport development

## Abstract

Within multi-level sport governance systems, community sport organizations (CSOs) can be understood as implementers of sport policy**.** In Canada, extensive research exists examining governance at the national level, and the managerial implications for clubs at the community level. However, there is a dearth of research on the role of governance/policy at the regional level. In this brief research report, we examine the role of CSOs as implementers of sport policy in Ontario, Canada. We used a case study methodology to answer two research questions: (1) How do actors from CSO's in Ontario understand their roles in developing sport participation opportunities? and, (2) How do CSOs’ institutional and community contexts shape organizational practices and the translation of ideas within sport development? We collected data through semi-structured interviews with 12 managers of CSOs affiliated with the same sport/provincial sport organization. Data were analyzed using thematic analysis. Findings indicate that CSOs have limited capacity for adaptation or change and that they experience powerful external pressures and competing demands. This research provides empirical insight into sport policy implementation processes in Ontario and highlights the importance of facility access as well as policy from other organizations/institutions (particularly municipalities and school boards) in shaping CSO practices.

## Introduction

Within federated multi-level sport governance systems, community sport organizations (CSOs) often serve as the primary mechanism through which sport participation opportunities are provided. Within these contexts, sport policy developed by national governments guides the work of organizations at the national, regional, and community level (1). In this way, CSOs can be understood as implementers of sport policy (2). However, in these institutionalized environments, organizational behaviours are shaped by a range of institutional factors (3, 4) as well as influences of the communities and regions in which they are embedded (5, 6).

Within Canada's multi-level governance system, extensive research exists examining governance at the national level [e.g., (7–9)], and the managerial implications for clubs at the community level [e.g., (10, 11)]. However, there is a dearth of research on the role of governance/policy at the regional level in Canada (6, 12). While policy is generally understood to flow from national to provincial and ultimately community level organizations, little research has interrogated the implementation of sport policy in Canada (13).

In this brief research report, we examine the role of CSOs as implementers of sport policy in Ontario, Canada. Specifically, we focus on the relationship between CSOs and regional bodies including their respective Provincial Sport Organization (PSO) and the provincial government. Framed as a case study analysis of policy implementation, we examined how policy moves through the sport system in Ontario. The research questions guiding this study were:

1. How do actors from CSO's in Ontario understand their roles in developing sport participation opportunities? and,
2. How do CSOs’ institutional and community contexts shape organizational practices and the translation of ideas within sport development?

## Context & literature review

CSOs in Ontario are largely non-profit organizations. While they are generally affiliated with a PSO, there are a growing number of commercial CSOs who deliver sport in an unsanctioned/unregulated manner. PSOs receive funding from the provincial government through the Ontario Amateur Sport Fund (14) and they support sport development and governance of CSOs in the province. While CSOs may qualify for and seek funding through federal, provincial, or municipal funding programs, the normally rely on subsidies and volunteer labour (1). Generally, CSOs pay fees to be a member of PSOs (15), who in turn support governance and coordinate development opportunities such as regional championships. CSOs in Ontario generally fund their operations by charging fees to members/participants and have little formal involvement with governments at the federal or provincial level. In many cases, municipalities support CSOs in other ways [e.g., providing free/low-cost facilities (16)]. CSOs rarely own and operate their own facilities and as such, rely heavily on other organizations (e.g., schools, municipalities, non-profits) for space.

From a neo-institutional perspective, scholars consider the structures and embedded agency of actors within sporting institutions (17). We draw on organizational institutionalism from a sociological perspective, considering institutions as “more-or-less taken-for-granted repetitive social behaviour that is underpinned by normative systems and cognitive understandings that give meaning to social exchange and thus enable self-reproducing social order” (18). We also acknowledge that institutions are “complex and often coherent mixtures of cultural and organizational material” (19, para. 27). As such, while governance and organizational structures frame their work, actors from CSOs are implicated in the translation of policy ideas within multi-level governance systems as they deliver sport participation opportunities in their communities. Translation is concerned with the socially constructed and performative nature of ideas related to policy as they move through sport systems (20). However, within translation processes, actors within different organizations shape how policy is understood and enacted. As such, actors may adapt or enact different versions of a similar idea that suits their club or community context (2, 21).

Multiple levels of governance and pronounced regional differences in Canada require scholars to acknowledge the complexity of institutional structures for CSOs. Greenwood et al. (3) described institutional complexity as a context in which organizations interact with multiple institutional logics and, as such face incompatible expectations or prescriptions. These logics are reflected in various field-level structures. Building on the work of Millar et al. (11), we were interested in field level structures related to the communities and regions in which CSOs are embedded, and how they shape organizational practices and translation. As noted by Marquis and Battilana (5), both the geographical context and organizational field have implications for understanding dynamics of change within institutions. As such, understanding how CSOs are embedded in their local communities is necessary to understand how policy flows in sport systems and ultimately, how decisions are made in CSOs (22–24). In particular, rural areas in Canada produce unique contexts that shape the way CSOs operate and engage with sport policy and governance systems (25, 26). For this work, we consider both how field-level structures (institutional pressures and logics) and community characteristics (local resources, infrastructure, and culture) shape the translation of ideas and the work of CSOs in Ontario.

Scholars have also highlighted how CSO capacity impacts the implementation of policy within sport systems. While human resources and financial capacity are repeatedly identified as important capacity dimensions for CSOs (27, 28), Hanlon et al. (21) identified how different capacity dimensions may be more salient for CSOs in pursuit of different kinds of policy goals. In an investigation of policy implementation related to the long-term athlete development model in Canada, Millar et al. (11) identified how the capacity of a particular CSO and poor communication between NSOs, PSOs, and CSOs shaped a haphazard process of translation characterized by both reproduction and adaptation.

In Canada, little scholarly inquiry has examined how ideas emanating from Canadian Sport Policy are translated through the sport system and ultimately shape governance and practice at the regional and provincial levels (13). Here, we build on our previous work (6) that examined the role of provincial government policy on organizational fields, and the implications it has for sport organizations. We approached this case study specifically to establish empirical insights to the factors that shape the translation and ultimately CSO behaviour in Ontario.

## Methodology

The present research project was part of a larger, multi-study research relationship with one PSO in Ontario, Canada. For this research, we used an instrumental case study methodology (29). The case examined is the translation of ideas between one (single sport) PSO and its constituent CSOs. Data were collected through semi-structured interviews with managers/board members (*n* = 12) from CSOs. Participants were recruited using a purposive sampling approach. Invitations were initially sent to all (*n* = 144) CSOs affiliated with the PSO. Follow up emails then targeted CSOs from specific regions of the province to obtain a sample that reflected the geographical makeup of affiliated CSOs (i.e., we specifically sought participants from all regions of the province). Based on discussions with partners at the PSO, we intentionally sought the perspectives of clubs with different organizational characteristics (e.g., large/small, professionalized/volunteer run, and primarily competition/recreational or social purpose focused). Interviews were conducted virtually, lasting between 23 and 79 min, and focused on participants work developing sport participation opportunities, their relationship with the PSO, and their understanding of policy and its impact on their organization. Data were analyzed using a six-step thematic analysis process (30) resulting in the development of two themes: (1) limited capacity for adaptation and change, and (2) external pressures and competing demands.

## Findings

In this section, we elaborate on the two themes developed. For each, we define their core meaning and substantiate with examples of data that best illustrate their intention.

## Limited capacity for adaptation and change

The first theme highlights the limitations that CSOs experienced due to their reliance on volunteer management teams, restricted resources, and limited facility access. Despite the generally positive reception of ideas and strategic guidance from their PSOs, CSOs struggled at times to implement changes due to a lack of operational capacity. Many actors described their organizations as operating at full capacity, with no room for additional members or programs.

The participant from CSO 12 noted that their greatest challenge to increasing participation within their community is access to “facilities and coaches.” They stated:

There’s a lot of demand, we’ll have 30 kids try out for a team and we can pick one team because we only end with gym time for one team and we only have one coach, whereas some other communities might then pick an A and B team and have them both … we just haven't been able to do that every time because we just don’t have any place for them.

Furthermore, a lack of access to facilities compounded these challenges, as organizations were often unable to meet their current members’ needs, let alone offer new participation opportunities. While many interviewees noted a desire to expand upon their programming to help meet the needs of a greater number of participants, this desire was not shared by all organizations. A participant from CSO 2 stated, “we're where we want to be and we're not looking to expand … we don't have the coaches or the facilities to expand our base, our base is just big enough for what we want to do.”

Participants noted that funding, access to facilities, and staffing support were their greatest needs to overcome these operational challenges. Many emphasized that their dependence on volunteers rather than paid staff prevented them from growing and adapting. This reliance limited organizations’ abilities to introduce new programs, accommodate more participants, or implement changes recommended by their PSO. As one respondent (CSO 1) noted, “the level of service that we can provide … and the number of teams that we can put out … is wholly dependent on the number of volunteers we get.” These constraints highlight a fundamental challenge in the sustainability and scalability of programs, where CSOs are unable to develop or diversify their programming to meet the needs of (often) growing and changing communities.

Within CSOs, the capacity to implement new ideas often depended on factors such as organizational size, access to resources, and the nature of their relationships with governing bodies. For larger organizations (often in or near larger population centres), alignment with PSO policies extended into strong, ongoing working relationships. These organizations often had regular communication with the PSO, benefiting from dedicated support, shared resources, and strategic guidance throughout the season. This close partnership allowed larger clubs to adopt and implement PSO-driven initiatives more effectively, as they received tailored support and could participate in joint planning efforts. One participant (from CSO 4, a large, urban club) mentioned that they were on a “first name, regular call basis” with the PSO. They further elaborated “we work hand in hand with them. Just because we are one of the biggest clubs, we always owe them some money and then it's like..we do a lot of hosting for them and help them run their league. So, we have a good relationship in that sense.”

Increased levels of professionalization within CSOs was also noted as a contributor to these issues. As a result of professionalization, participants identified a greater burden on volunteer coaches, in particular to complete rigorous certification and training. As noted by the actor from CSO 11:

The other thing we struggle with is constantly getting volunteers who want to put in the time and get certified. I mean that’s yearly you got to take the courses…and they take the courses and then..They don't finish the coursework afterwards. They'll take the initial part and it’s and it’s really hard because they're they have full-time jobs, they have families. They're young.

Another constraint within this theme involved the challenges related to facility access, particularly at local schools. Many CSOs rely on school facilities to run their programming and therefore must navigate complex scheduling demands and changing priorities of school boards. CSOs were often competing for the space with other organizations. This competition for limited facility access intensified capacity struggles within organizations. The participant from CSO 1 noted that their hold upon space is often precarious as “[schools] have a long list of Girl Guides and Boy Scouts and all sorts of different organizations that are outside of sport … with interest in using the same facilities that you have to compete with for facility time.”

## External pressures and competing demands

The second theme represents actors’ understandings of the multiple and competing pressures that shaped organizational practices related to sport development. While the PSO provided important resources related to coaching and insurance coverage, local municipalities and school board policies exerted major influence on CSOs as they provided important resources, most notably the physical spaces for sport participation. Additionally, changing administrative requirements passed down from governments and governing bodies caused CSOs to dedicate increasing amounts of time and human resources towards club administration. These pressures resulted in CSOs experiencing tensions related to the fulfillment of administrative priorities not related to sport.

An important pressure shaping organizational practices came from the policy of local municipalities and school boards, as these external stakeholders largely controlled access to facilities. The tenuous grasp on participation locations was felt acutely during disruptions caused by the COVID-19 pandemic as schools closed their spaces across the province. The participant from CSO 9 stated that “one of the things that COVID showed us is that our access to gyms was at the mercy of the school boards and if they decided to close because of COVID, there wasn't much we could do”

A reliance on school boards and municipal authorities for gymnasiums and other recreational spaces often placed CSOs at the mercy of shifting local policies and competing demands for facility access. Changes in school board or municipal priorities could directly impact the availability of spaces necessary for sport programming. The participant from CSO 12 reported that due to a lack of municipal facilities in their region, the years following the COVID pandemic were increasingly difficult to navigate as “the schools and post-secondary institutions were really reluctant to allow community groups back in and it was a horrible policy decision for children.” This participant also called for increased accountability in provincial legislation:

That [community use of school policy] mandates school boards to make their facilities available to community users … these are publicly funded infrastructure … they should be available when they're not being used by that school or that school board. But that’s not how it actually plays out. And there’s not, as far as I can see, there’s no oversight and accountability to these schools and school boards when they don't make their facilities available.

The dependence on external organizations for facilities intensified the challenges of meeting participation targets and maintaining quality of programs.

Despite the challenges they experienced, many organizations also expressed an understanding of, and commitment to the overarching goals set by their PSOs, recognizing the value of policies and procedures that were presented to them from organizations above them within the multi-level governance structure. When asked about how their organization aligns with policy from their governing body, the actor from CSO 1 stated that:

We absolutely will align with for example, Rowan’s law [concussion safety] … [the PSO] has made that requirement, and we will align with that requirement because it makes sense, right? [The PSO] has fair play rules that you have to sign off on, and we will make sure that every coach and every player signs off on fair play rules. So, yeah, absolutely. Like we will 100% align with what our provincial organization requires.

In contrast, smaller clubs and organizations in rural areas had more limited interactions with their PSO, with some only engaging with them at the beginning of the season to coordinate insurance coverage and membership numbers. These CSOs reported little ongoing communication or support, which left them feeling more disconnected from PSO initiatives and often struggling to independently manage the demands of policy implementation. As a result, these smaller organizations were often left to interpret and apply policies on their own based on the information provided through the PSO website. Some clubs noted the increased burden of policy changes, especially those that required training or endorsement from boards/participants as something that they struggled with as volunteer-run organizations. The actor from CSO 3 noted that:

They [the PSO] provide a structure as far as coaching goes, they are playing an important role. However, I do feel that they keep adding more and more layers of administrative roles for clubs … we're all volunteer-led organizations [and we] are all giving our time for this … the more time that’s required for off court things, it takes away the time that we can go on court for the actual participation.

Additionally, some clubs located outside the Greater Toronto Area (GTA) perceived the PSO as being primarily focused on GTA-based clubs, which created a sense of underrepresentation for those in other regions. These non-GTA organizations felt that the PSO's programs, events, and policy priorities were disproportionately directed toward the needs of urban/GTA clubs, making it difficult for clubs outside of this region to engage fully in competition due to the travel and resource demands. One participant, from CSO 9 (based in Eastern Ontario) mentioned that the PSO “tends to be, unfortunately, a very Toronto-centric organization. I believe all of their board members are from the GTA, so that can be problematic.”

Another challenge for CSOs was the need to comply with evolving administrative requirements, such as the requirement to incorporate as a non-profit within the Province of Ontario, which was enforced in late 2024. Many participants expressed difficulty in navigating this incorporation process, which involved legal, financial, and operational adjustments that volunteer-led organizations were ill-equipped to handle. For volunteer-run CSOs, becoming a registered non-profit required additional paperwork, compliance with provincial regulations, and an understanding of governance structures, which proved to be a burden on their limited resources. The participant from CSO 6 noted how these changes impacted their organization: “this past year they changed the rules around what constituted a not-for-profit organization and so then we had to go and adjust all of our bylaws and all of our policies to maintain our not-for-profit status.”

In a changing and complex environment, CSOs found themselves balancing multiple, sometimes competing demands. For many organizations, these demands had implications for how they understood and engaged with policy directives and how they engaged with the PSO.

## Discussion

Collectively, this case study illustrates the complexity faced by CSOs as they navigated a range of institutional factors that influence the translation of ideas and implementation of sport policy in Ontario. The case study contributes empirical insights into sport policy implementation processes in Canada. Here, we identify these contributions and their implications for both theory and practice.

Broadly, our findings align with research that has highlighted the importance of organizational capacity and communication between NSOs, PSOs, and CSOs within Canadian multilevel governance system [e.g., (1, 11)]. Building on this work, our findings illustrate how communication may be implicated within broader, existing relationships between these organizations. Characteristics such as size, level of professionalization, and geographic location were identified as factors implicated in shaping the relationships between CSOs and their PSO. These characteristics had implications for how participants understood their club, their role in developing sport participation opportunities, and by extension, how ideas were translated into practice within CSOs. Future research and practice should consider the implications of these organizational characteristics specifically and how they shape policy implementation processes.

Previous research also suggests that different dimensions of capacity may be more salient considerations for organizations in pursuing different outcomes or policy directives (21). While our findings aligned with previous work [e.g., (27, 28)] by indicating that finances and human resources were important capacity issues for CSOs in Ontario, participants also indicated that access to facilities was a very salient factor that limited their ability to expand or adapt their programming. These capacity issues are implicated in their broader institutional contexts and reflect how provincial policy [as discussed by (6)] impacts sport organizations in Ontario. As CSOs in Ontario (and Canada more broadly) rarely have their own facilities, they rely on the use of municipal and school facilities, which squarely embeds CSOs in the politics of local municipalities and school boards (and by extension provincial governments). Therefore, our analysis illustrates the impacts of provincial policy in these related fields and highlights a need to consider the alignment of policy across public institutions, and how (mis)alignment impacts CSOs. As such, future research should examine the role of municipal and school board policy in shaping CSO practices and the implementation of sport policy in diverse geographical contexts as well as for different sports. Future work may adopt an approach similar to Hanlon et al. (21) in examining policy focused on a specific issue (in their case gender equity in sport) or look more broadly at administrative practices and approaches adopted within these allied public organizations and their implications for sport development practices and policy implementation.

Finally, our findings also illustrate the complexity of the institutional context in which CSOs operate. Participants’ experiences demonstrate how institutional structures (from fields associated with sport, education, and municipalities) as well as their community contexts (e.g., geography, availability of human resources and facilities, etc.) may shape the translation of ideas within their organizations. These findings contribute to the literature [e.g., (22–24)] that has begun to map out the implications of community embeddedness for sport organizations and policy implementation. Building on our findings, future work may seek to map out the different processes through which local communities inform logics that underpin decision making in CSOs, and how they shape the adaptation of policy ideas within translation processes.

## Conclusion

In this brief research report, we used a case study to explore sport policy implementation processes in Ontario, Canada. Specifically, we examined how actors from CSOs understood their role in developing sport participation opportunities, and the role of institutional and community contexts in shaping the translation of ideas and implementation of policy in the province. In focusing on multiple CSOs from one sport/PSO within the province, we offered a novel unit of analysis. While efforts were made to recruit participants from clubs of different sizes and regions, a limitation of the small sample is that participants’ experiences likely do not reflect the diversity of CSOs and geographical contexts in which CSOs operate. Further, our analysis reflects only one sport and, as such certain policy and governance issues (e.g., access to school facilities) may not be uniform across different sports.

Future research should continue to examine the complexity of these processes across sports, community contexts, and administrative jurisdictions (i.e., provinces and territories). While we examined the perspectives of CSO-affiliated actors in this research, participants highlighted how a variety of actors from the public and private sectors were implicated in sport development processes. Future work should examine the role and agency of other actors who are underrepresented in the literature from an institutional work perspective (17). In Ontario, this research may focus on PSOs, municipalities, and the Provincial Government to understand how they may be seeking out social and political change. Moving forward, a robust understanding of sport policy implementation in Canada will require examination of the complex interplay (including both synergies and incongruencies) of policy at the municipal, provincial, and federal level as well as case studies that examine the successes and challenges of developing sport participation opportunities in different jurisdictions across the country.

## Data Availability

The datasets presented in this article are not readily available because data collected will remain anonymous and confidential to everyone but the research team. Requests to access the datasets should be directed to krich@brocku.ca.
